# A Myristoyl Amide Derivative of Doxycycline Potently Targets Cancer Stem Cells (CSCs) and Prevents Spontaneous Metastasis, Without Retaining Antibiotic Activity

**DOI:** 10.3389/fonc.2020.01528

**Published:** 2020-09-15

**Authors:** Béla Ózsvári, Luma G. Magalhães, Joe Latimer, Jussi Kangasmetsa, Federica Sotgia, Michael P. Lisanti

**Affiliations:** ^1^Translational Medicine, School of Science, Engineering and Environment (SEE), University of Salford, Manchester, United Kingdom; ^2^Salford Antibiotic Research Network, School of Science, Engineering and Environment (SEE), University of Salford, Manchester, United Kingdom; ^3^Eurofins Integrated Discovery UK Ltd., Essex, United Kingdom; ^4^Lunella Biotech, Inc., Ottawa, ON, Canada

**Keywords:** cancer stem-like cells (CSCs), Doxycycline, myristic acid, fatty acylation, cancer cell metastasis, prophylaxis of metastasis, 9-amino-Doxycycline, antimitoscins

## Abstract

Here, we describe the chemical synthesis and biological activity of a new Doxycycline derivative, designed specifically to more effectively target cancer stem cells (CSCs). In this analog, a myristic acid (14 carbon) moiety is covalently attached to the free amino group of 9-amino-Doxycycline. First, we determined the IC_50_ of Doxy-Myr using the 3D-mammosphere assay, to assess its ability to inhibit the anchorage-independent growth of breast CSCs, using MCF7 cells as a model system. Our results indicate that Doxy-Myr is >5-fold more potent than Doxycycline, as it appears to be better retained in cells, within a peri-nuclear membranous compartment. Moreover, Doxy-Myr did not affect the viability of the total MCF7 cancer cell population or normal fibroblasts grown as 2D-monolayers, showing remarkable selectivity for CSCs. Using both gram-negative and gram-positive bacterial strains, we also demonstrated that Doxy-Myr did not show antibiotic activity, against *Escherichia coli* and *Staphylococcus aureus*. Interestingly, other complementary Doxycycline amide derivatives, with longer (16 carbon; palmitic acid) or shorter (12 carbon; lauric acid) fatty acid chain lengths, were both less potent than Doxy-Myr for the targeting of CSCs. Finally, using MDA-MB-231 cells, we also demonstrate that Doxy-Myr has no appreciable effect on tumor growth, but potently inhibits tumor cell metastasis *in vivo*, with little or no toxicity. In summary, by using 9-amino-Doxycycline as a scaffold, here we have designed new chemical entities for their further development as anti-cancer agents. These compounds selectively target CSCs, e.g., Doxy-Myr, while effectively minimizing the risk of driving antibiotic resistance. Taken together, our current studies provide proof-of-principle, that existing FDA-approved drugs can be further modified and optimized, to successfully target the anchorage-independent growth of CSCs and to prevent the process of spontaneous tumor cell metastasis.

## Introduction

Cancer stem cells (CSCs) are thought to be the root cause of recurrence, metastasis, and drug-resistance, in a host of cancer types (1–5). As a consequence, there is an unmet need to develop new therapeutics to target and selectively kill CSCs, while avoiding side effects, especially severe chemo-toxicity. Metastasis is believed to be driven by this unique sub-population of cancer-initiating cells (1–3). CSCs have the ability to generate *de novo* tumors in immuno-deficient host organisms. Moreover, they have the capacity to engage in anchorage-independent growth, which facilitates their invasive spread throughout the various tissues and organ systems, resulting in local, and distant disseminated lesions (4, 5). Remarkably, these metastatic lesions are resistant to both chemo-therapy and radiation treatments. Unfortunately, the Achilles' heel of these pro-metastatic CSCs remains largely unknown. As a consequence, currently there are no anti-cancer drugs that are FDA-approved for the prevention of metastasis.

Over the last 5 years, we identified that mitochondria in CSCs may be a novel tractable therapeutic target, for inhibiting their anchorage-independent growth (5). More specifically, increased mitochondrial biogenesis may facilitate efficient high energy production, resulting in the rapid propagation of CSCs (6–10). In addition, within metastatic lymph nodes isolated from breast cancer patients, disseminated cancer cells show elevated levels of mitochondrial activity, especially Complex IV activation, as revealed by functional activity assays (11).

Interestingly, mitochondrial biogenesis is critically linked to the activity of mitochondrial ribosomal proteins, that functionally translate key mitochondrial proteins, which are genetically encoded by mitochondrial DNA (mt-DNA); this includes 13 proteins that are necessary to functionally maintain OXPHOS and mitochondrial ATP synthesis (6–13).

The reason that mitochondria have their own DNA and specific machinery for protein translation is that they originally evolved from engulfed aerobic bacteria, over the last 1.4 billion years, after invading eukaryotic cells. This evolutionary symbiotic relationship has certain functional consequences that could be safely exploited to achieve anti-mitochondrial therapy, specifically targeting CSCs. For example, because of the similarities between bacteria and mitochondria, a sub-set of bacteriostatic antibiotics block mitochondrial protein translation, as a manageable side effect (6, 10, 13). In this context, Doxycycline inhibits the activity of the small mito-ribosome, while Azithromycin blocks the large mito-ribosome, both as off-target side effects (6, 10, 13). Similarly, Doxycycline and Azithromycin both inhibit the propagation of CSCs, in an anchorage-independent fashion, in numerous breast cancer cell lines, as well as in cell lines derived from many other solid tumor types (6, 10, 13). As such, we have suggested that these side-effects could be re-purposed to clinically target and therapeutically eradicate CSCs.

In direct support of this notion, a Phase II clinical trial has documented that brief treatment with Doxycycline, in early breast cancer patients, is indeed sufficient to significantly decrease the content of CSCs in the tumor mass, by employing CD44-staining as an established marker of CSCs in ER(+) patients (14). Overall, the response rate approached 90%, resulting in reductions of up to nearly 67% in CSC tumor burden (14). Quantitatively similar results were obtained in HER2(+) patients, using ALDH1 as a CSC marker. As such, blocking mitochondrial protein synthesis may be a viable approach for targeting and removing CSCs *in vivo*, prior to surgical excision, possibly preventing the development of metastases.

Consistent with the above observations, other groups have shown that Doxycycline treatment effectively reduces the expression of a panel of CSC markers, in breast cancer cell lines, such as CD44, ALDH, Oct4, Sox2, and Nanog (9, 12). Moreover, Doxycycline treatment functionally inhibits multiple CSC signaling pathways, including Wnt, Notch, Hedgehog, and STAT1/3-signaling (10).

Here, we describe a medicinal chemistry approach to design novel therapeutics to more selectively target CSCs. More specifically, we show that several new potent Doxycycline analogs can be generated by attaching a fatty acid onto 9-amino-Doxycycline, which is a relatively straightforward chemical modification. Importantly, these analogs, such as Doxy-Myr, lack antibiotic activity, but more potently target CSCs and effectively prevent metastasis, in an *in vivo* pre-clinical model. Therefore, Doxy-Myr could be used to eradicate CSCs, without exerting the selective pressures required for the development of antimicrobial resistance and without significant toxicity.

To better describe this general class of novel compounds which are lipid-modified FDA-approved antibiotics, we propose the term Antimitoscins, to specifically reflect their intrinsic anti-mitochondrial activity.

## Materials and Methods

### Materials

MCF7 and MDA-MB-231 cells were obtained from the American Type Culture Collection (ATCC). hTERT-BJ1 fibroblasts were as we previously described (13). Cells were cultured in DMEM, supplemented with 10% fetal calf serum (FCS), Glutamine and Pen/Strep.

### Chemical Synthesis

Custom-chemical syntheses were performed by Eurofins Integrated Discovery UK Ltd., (Essex, UK). Conventional peptide synthesis methods were used to covalently attach each free fatty acid to 9-amino-Doxycycline. The desired reaction products were identified, chromatographically purified and the chemical structures were validated, by using a combination of NMR and mass spectrometry. The IUPAC names for the chemical compounds are as follows.

#### Doxycycline

(4S,5S,6R,12aS)-4-(dimethylamino)-3,5,10,12,12a-pentahydroxy-6-methyl-1,11-dioxo-4a,5,5a,6-tetrahydro-4H-tetracene-2-carboxamide.

#### Doxycycline-Myr

(4S,5S,6R,12aS)-4-(dimethylamino)-3,5,10,12,12a-pentahydroxy-6-methyl-1,11-dioxo-9-(tetradecanoylamino)-4a,5,5a,6-tetrahydro-4H-tetracene-2-carboxamide.

#### Doxycycline-Laur

(4S,5S,6R,12aS)-4-(dimethylamino)-9-(dodecanoylamino)-3,5,10,12,12a-pentahydroxy-6-methyl-1,11-dioxo-4a,5,5a,6-tetrahydro-4H-tetracene-2-carboxamide.

#### Doxycycline-Pal

(4S,5S,6R,12aS)-4-(dimethylamino)-9-(hexadecanoylamino)-3,5,10,12,12a-pentahydroxy-6-methyl-1,11-dioxo-4a,5,5a,6-tetrahydro-4H-tetracene-2-carboxamide.

#### Doxycycline-TPP

[6-[[(5R,6S,7S,10aS)-9-carbamoyl-7-(dimethylamino)-1,6,8,10a,11-pentahydroxy-5-methyl-10,12-dioxo-5a,6,6a,7-tetrahydro-5H-tetracen-2-yl]amino]-6-oxo-hexyl]-triphenyl-phosphonium oxalate.

#### 9-Amino-Doxycycline

(4S,5S,6R,12aS)-9-amino-4-(dimethylamino)-3,5,10,12,12a-pentahydroxy-6-methyl-1,11-dioxo-4a,5,5a,6-tetrahydro-4H-tetracene-2-carboxamide.

9-Amino-Doxycycline was synthesized, essentially as previously described (15).

See Supplemental Materials and Methods, and Supplemental Figure 1, for further details on the chemical synthesis of these Doxycycline derivatives.

### 3D-Mammosphere Assay

The mammosphere assay is considered as the gold-standard for functionally measuring “stemness” and CSC propagation in breast cancer cells. Single cells are first plated at low density on low-attachment plates and >90% of “bulk” cancer cells die under these conditions, in a process of programmed cell death, termed anoikis. Only stem-like cells survive and propagate in suspension. Each 3D mammosphere is formed from a single CSC. Test compounds are added at the moment of single cell plating and then the number of 3D mammospheres are counted 5 days after plating. More specifically, a single cell suspension of MCF7 cells was prepared using enzymatic (1x Trypsin-EDTA, Sigma Aldrich) and manual disaggregation (25-gauge needle) (16–19). Cells were then plated at a density of 500 cells/cm^2^ in mammosphere medium (DMEM-F12/B27/ EGF (20-ng/ml)/PenStrep) in non-adherent conditions, in culture dishes coated with (2-hydroxyethylmethacrylate) (poly-HEMA, Sigma). Cells were then grown for 5 days and maintained in a humidified incubator at 37°C at an atmospheric pressure in 5% (v/v) carbon dioxide/air. After 5 days in culture, spheres >50 μm were counted using an eye-piece graticule, and the percentage of cells plated which formed spheres was calculated and is referred to as percent mammosphere formation, normalized to vehicle-alone treated controls. Mammosphere assays were performed in triplicate and repeated three times independently.

### Fluorescence Imaging

Fluorescent images were taken after 72 h of incubation of MCF7 cells treated with either Doxycycline or Doxy-Myr (both at 10 μM), or vehicle control. Cell cultures were imaged with the EVOS Cell Imaging System (Thermo Fisher Scientific, Inc.), using the GFP channel. No fluorescent dye was used before imaging, therefore, any changes in signal were exclusively due to the auto-fluorescent nature of the Doxycycline compounds.

### Cell Viability Assay

The Sulphorhodamine (SRB) assay is based on the measurement of cellular protein content (16–19). After treatment for 72 h in 96-well plates (8,000 cells/well), cells were fixed with 10% trichloroacetic acid (TCA) for 1 h in the cold room, and were dried overnight at room temperature. Then, cells were incubated with SRB for 15 min, washed twice with 1% acetic acid, and air dried for at least 1 h. Finally, the protein-bound dye was dissolved in a 10 mM Tris, pH 8.8, solution and read using the plate reader at 540-nm.

### Cell Proliferation

Briefly, MCF7 cells were seeded in each well (10,000 cells/well) and employed to assess the efficacy of Doxycycline and Doxy-Myr, using RTCA (real-time cell analysis), via the measurement of cell-induced electrical impedance plate (Acea Biosciences Inc.) (18). This approach allows the quantification of the onset and kinetics of the cellular response. Experiments were repeated several times independently, using quadruplicate samples for each condition.

### Cell Cycle Analysis

We performed cell-cycle analysis on MCF7 cells treated with Doxycycline, Doxy-Myr, or vehicle-alone. Briefly, after trypsinization, the re-suspended cells were incubated with 10 ng/ml of Hoechst solution (Thermo Fisher Scientific) for 40 min at 37°C under dark conditions. Following a 40 min period, the cells were washed and re-suspended in PBS Ca/Mg for acquisition on the Attune NxT flow cytometer (Thermo Scientific). We analyzed 10,000 events per condition. Gated cells were manually-categorized into cell-cycle stages (19).

### Bacterial Growth Assays

Briefly, antibiotic activity was assessed using standard assay systems (20–22). The antibiotic activity of Doxycycline analogs was determined experimentally, using Resazurin (R7017; Sigma-Aldrich, Inc.) as an indicator of bacterial metabolism/vitality, in a 96-well plate format, using *Escherichia coli* and *Staphylococcus aureus*. The minimum inhibitory concentration (MIC) for the studied compounds was determined using the broth microdilution method, the reference susceptibility test for rapidly growing aerobic or facultative microorganism(s). The assays were performed according to the Clinical and Laboratory Standards Institute (CLSI) guidelines. The test compounds and positive control (doxycycline, Sigma Aldrich #D1822) stock solutions were prepared at 25 mM in DMSO and serially diluted (2-fold dilution from 200 to 1.56 μM) in cation adjusted Mueller Hinton Broth (MHB, Sigma Aldrich #90922) in 96-well transparent plates (VWR #734-2781) into a final volume of 50 μL/well. *Staphylococcus aureus* (ATCC 29213) and *E. coli* (ATCC 25922) cultures were maintained on Mueller Hinton Agar (MHA, Sigma Aldrich #70191). A single colony of each strain was then grown overnight at 37 °C in MHB until OD_600_ ~ 0.6–0.8 and diluted into MHB to a concentration of 106 colony forming units (CFU)/mL, which was equivalent to an OD_600_ ~ 0.01. Diluted inocula (50 μL) were transferred to the wells of the previously prepared 96-well plates containing the test compounds, negative control (1% DMSO in MHB) and positive control (Doxycycline). Final wells volume was 100 μL, final concentrations for the testing compounds were between 100 and 0.78 μM and final microorganism concentration was 5 × 10^5^ CFU/mL. Subsequently, 10 μL of one negative control well was plated in a petri dish containing MHA to check CFU and the purity of the cultures. The plates were incubated at 37°C for 24 h after which 20 μL of resazurin solution (0.2 mg/mL) was added to the wells followed by 1 h 30 min incubation at 37°C. The OD_570_ and OD_600_ were measured in a microplate reader (BMG FLUOstar Omega). The ratio between OD_570_ and OD_600_ was determined and the MIC represents the lowest concentration of compound that inhibited bacterial growth (OD_570_/OD_600_ ratio inferior to the average ratio determined for negative control wells). MIC values were determined by three independent experiments.

### Assays for Tumor Growth, Metastasis, and Embryo Toxicity

These xenograft assays were carried out, essentially as previously described, without any major modifications (23–27).

#### Preparation of Chicken Embryos

Fertilized White Leghorn eggs were incubated at 37.5°C with 50% relative humidity for 9 days. At that moment (E9), the chorioallantoic membrane (CAM) was dropped down by drilling a small hole through the eggshell into the air sac, and a 1 cm^2^ window was cut in the eggshell above the CAM.

#### Amplification and Grafting of Tumor Cells

The MDA-MB-231 tumor cell line was cultivated in DMEM medium supplemented with 10% FBS and 1% penicillin/streptomycin. On day E9, cells were detached with trypsin, washed with complete medium and suspended in graft medium. An inoculum of 1 × 10^6^ cells was added onto the CAM of each egg (E9) and then eggs were randomized into groups.

#### Tumor Growth Assays

At day 18 (E18), the upper portion of the CAM was removed from each egg, washed in PBS and then directly transferred to paraformaldehyde (fixation for 48 h) and weighed. For tumor growth assays, at least 10 tumor samples were collected and analyzed per group (*n* ≥ 10).

#### Metastasis Assays

On day E18, a 1 cm^2^ portion of the lower CAM was collected to evaluate the number of metastatic cells in 8 samples per group (*n* = 8). Genomic DNA was extracted from the CAM (commercial kit) and analyzed by qPCR with specific primers for Human Alu sequences. Calculation of Cq for each sample, mean Cq, and relative amounts of metastases for each group are directly managed by the Bio-Rad® CFX Maestro software. A one-way ANOVA analysis with post-tests was performed on all the data.

#### Embryo Tolerability Assay

Before each administration, the treatment tolerability was evaluated by scoring the number of dead embryos.

### Statistical Analysis

Statistical significance was determined using the Student's *t*-test, values of <0.05 were considered significant. Data are shown as the mean ± SEM, unless stated otherwise. Also, ANOVA was conducted, where appropriate.

## Results

### Generating New Analogs of Doxycycline for Targeting CSCs: Doxy-Myr and Doxy-TPP

Doxycycline is known to function as an inhibitor of the propagation of CSCs, through its ability to inhibit the small mitochondrial ribosome, which is an off-target side-effect (6, 10, 13). Normally, Doxycycline is used as a broad-spectrum bacteriostatic antibiotic to treat a wide range of infections caused by gram-negative and gram-positive bacteria. Therefore, we sought to optimize the ability of Doxycycline for the targeting of CSCs, while minimizing its antibiotic activity, to derive a new chemical entity to selectively target CSCs.

**Figure 1:**
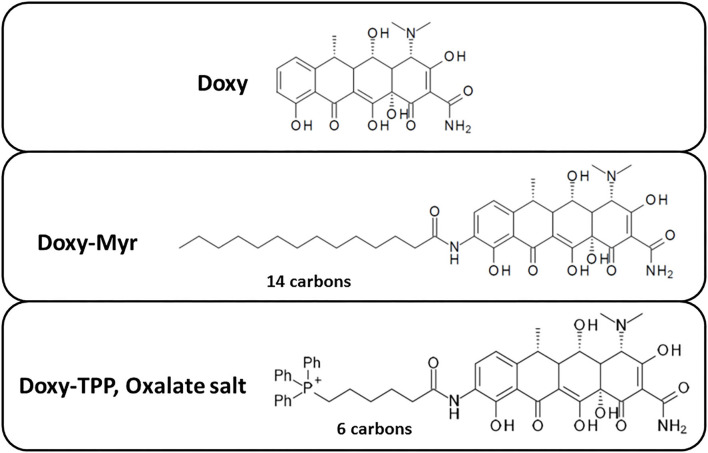
Chemical structures of two new Doxycycline derivatives: Doxy-Myr and Doxy-TPP. Note that Doxy-Myr contains a 14-carbon fatty acid (myristate) covalently attached to 9-amino-Doxycycline and Doxy-TPP contains a TPP-moiety attached via a 6-carbon spacer to 9-amino-Doxycycline.

As a first step, we synthesized 9-amino-Doxycycline, to which we covalently attached either a 14 carbon fatty acid moiety (myristic acid) or a six carbon spacer arm containing tri-phenyl-phosphonium (TPP). The chemical structures of these two Doxycycline analogs, as well as the parent compound, are all shown in Figure 1. We speculated that the TPP-moiety would better target Doxy-TPP to mitochondria in CSCs. In contrast, the addition of myristic acid could act as a membrane targeting signal, possibly leading to the increased retention of Doxy-Myr within membranous compartments, such as the plasma membrane, the endoplasmic reticulum (ER), the Golgi apparatus, and/or mitochondria.

To determine the functional activity of these Doxycycline analogs, we used the 3D-mammosphere assay, to assess their ability to inhibit the 3D anchorage-independent propagation of MCF7 CSCs. Interestingly, Doxy-TPP was not more potent that Doxycycline itself, so further assays with Doxy-TPP were not carried out (data not shown).

**Figure 2:**
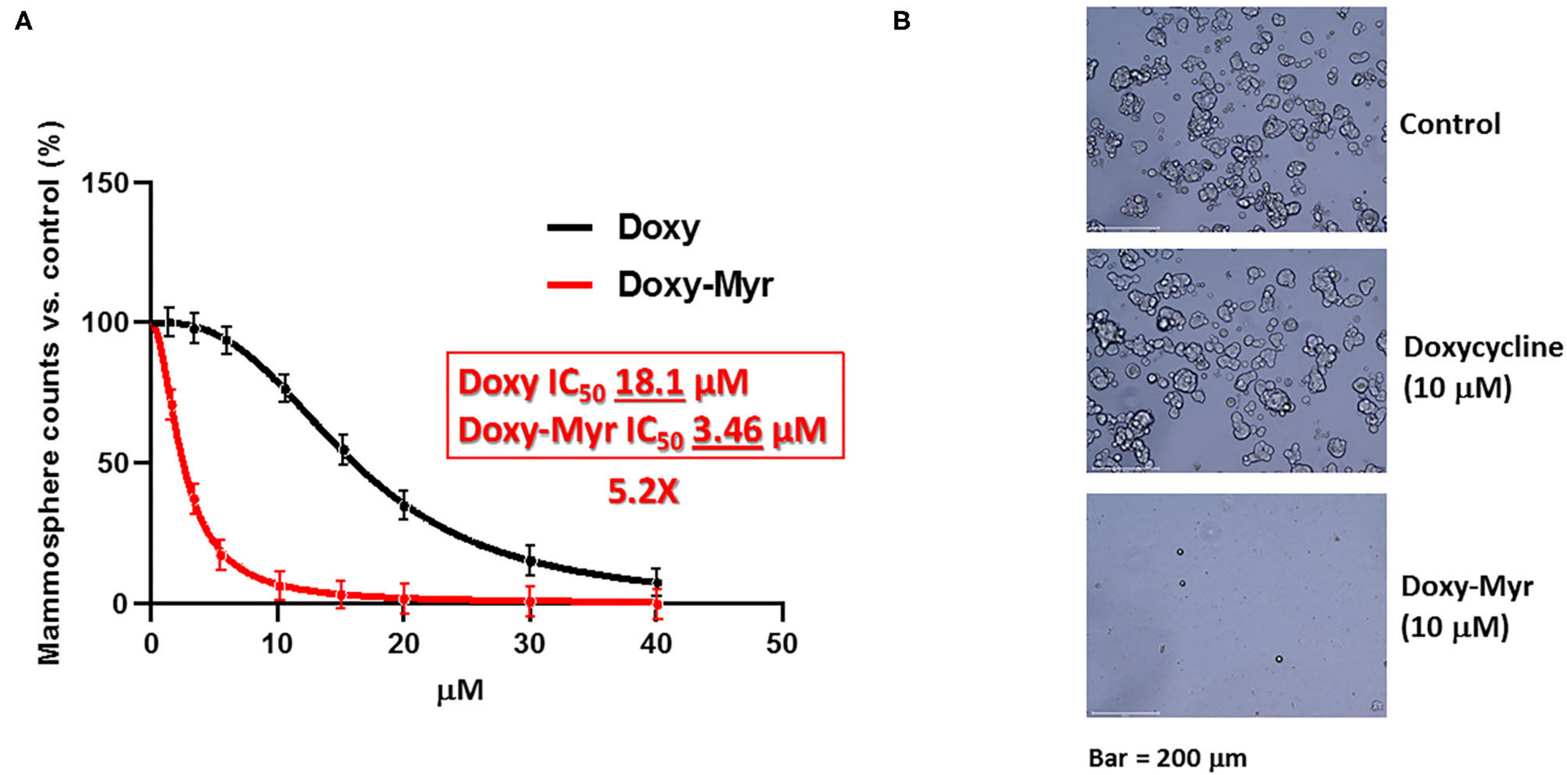
Doxy-Myr more potently inhibits the anchorage-independent propagation of CSCs. **(A)** MCF7 cells were plated under anchorage-independent growth conditions and the number of 3D-mammospheres were counted after 5 days. Note that Doxycycline and Doxy-Myr both inhibited 3D-mammosphere formation, all relative to vehicle-alone controls. However, Doxy-Myr was >5-fold more potent than Doxycycline (IC-50 of 3.46 vs. 18.1 μM). **(B)** Representative phase images of 3D-mammospheres are shown. Bar = 200 μm.

Figure 2 shows a direct comparison of Doxycycline with Doxy-Myr. Remarkably, Doxy-Myr was >5-fold more potent than Doxycycline, with an IC_50_ of 3.46 μM. In contrast, Doxycycline had an IC_50_ of 18.1 μM. Therefore, Doxy-Myr is more potent for targeting the 3D anchorage-independent propagation of CSCs.

**Figure 3:**
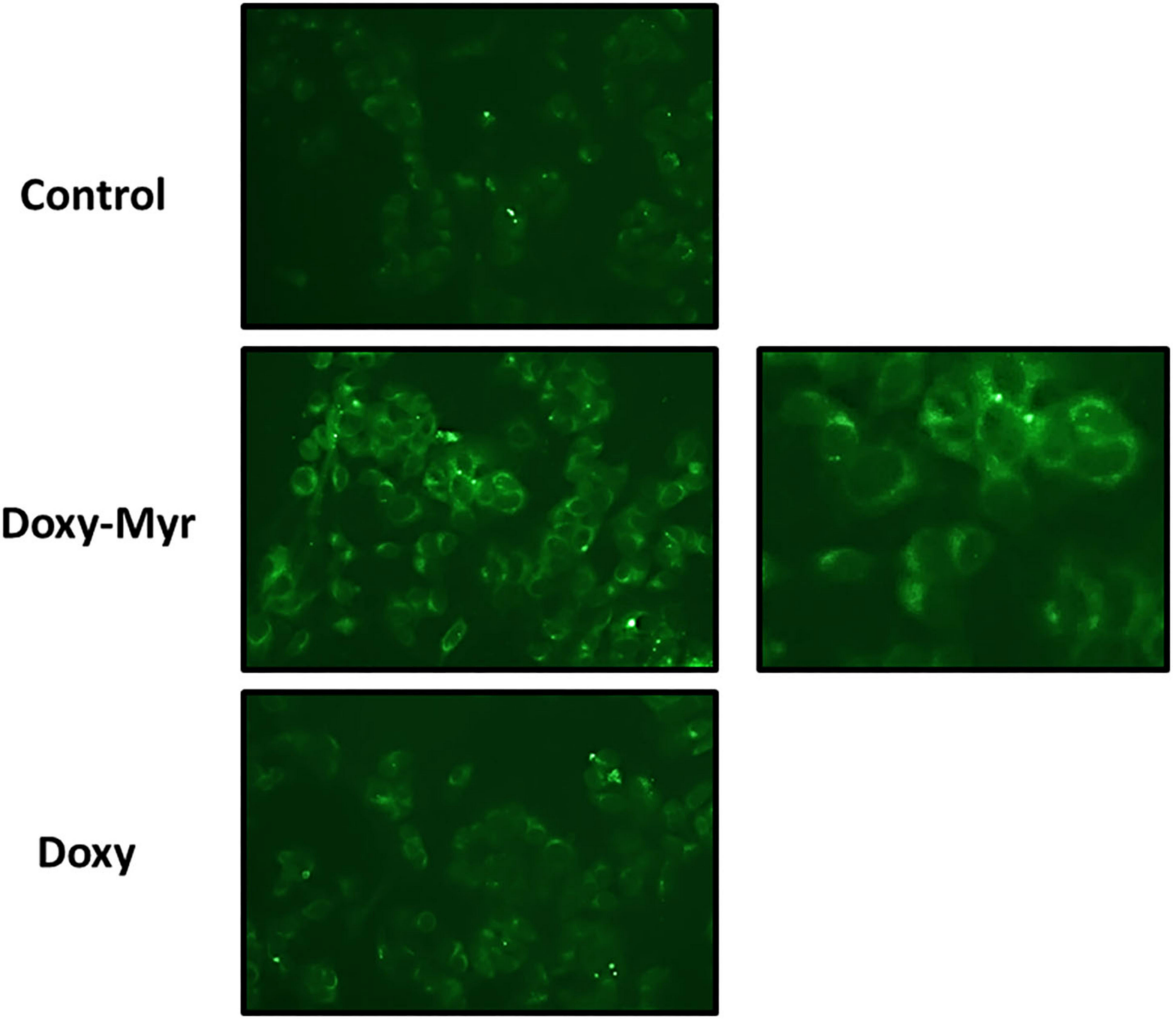
Doxy-Myr is better retained within cells and reveals a peri-nuclear staining pattern. MCF7 cells were cultured for 72 h as 2D-monolayers, in the presence of Doxycycline or Doxy-Myr, at a concentration of 10 μM. Vehicle-alone controls were processed in parallel. Then, MCF7 cells were washed twice with PBS and subjected to live cell imaging to capture the auto-fluorescent signal retained within cells. Note that Doxy-Myr showed the strongest intracellular retention, and was concentrated within peri-nuclear intracellular compartments. No nuclear staining was observed. Quantitation of mean pixel intensity, using Image J software, revealed that relative to Doxycycline, Doxy-Myr showed a near 3-fold increase in intracellular fluorescence.

To experimentally test the hypothesis that Doxy-Myr was better retained within cells, we took advantage of the observation that Doxycycline is fluorescent (Ex. 390–425 nm/Em. 520–560 nm). Doxy-Myr was more easily detected and retained in monolayer MCF7 cells, when compared to Doxycycline or cells treated with vehicle alone (Figure 3). Doxy-Myr fluorescence showed a peri-nuclear staining pattern, consistent with its partitioning and retention within intracellular membranous compartments. This observation could mechanistically explain its increased potency. No nuclear staining for Doxy-Myr was observed, indicating that it was predominantly excluded from the nucleus.

### Doxy-Myr Is Non-toxic in 2D-Monolayers of MCF7 Cells and Normal Human Fibroblasts

**Figure 4:**
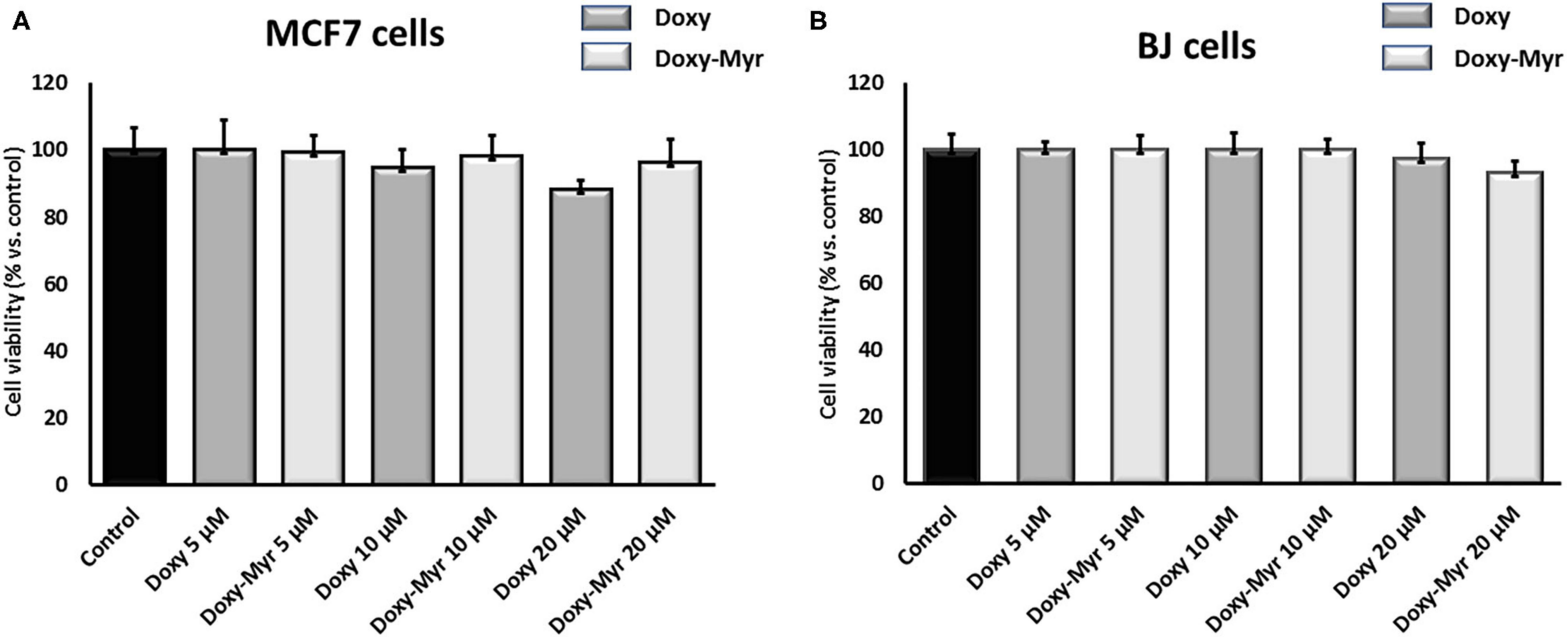
Doxy-Myr does not affect the viability of MCF7 cells or normal fibroblasts, when grown as 2D-monolayers. To determine the effects of Doxy-Myr on cell viability, we next treated MCF7 cells and normal human fibroblasts (hTERT-BJ1) over a period of 3 days. Note that Doxycycline and Doxy-Myr had no appreciable effects on cell viability, in the concentration range of 5–20 μM. **(A)** MCF7 cells, **(B)** hTERT-BJ1 cells.

To further assess the effects of Doxy-Myr on 2D-cell growth, we next used MCF7 cells and normal human fibroblasts (hTERT-BJ1) treated over a period of 3 days. Figure 4 shows that both Doxycycline and Doxy-Myr had no appreciable effects on cell viability, as determined over the concentration range of 5–20 μM.

**Figure 5:**
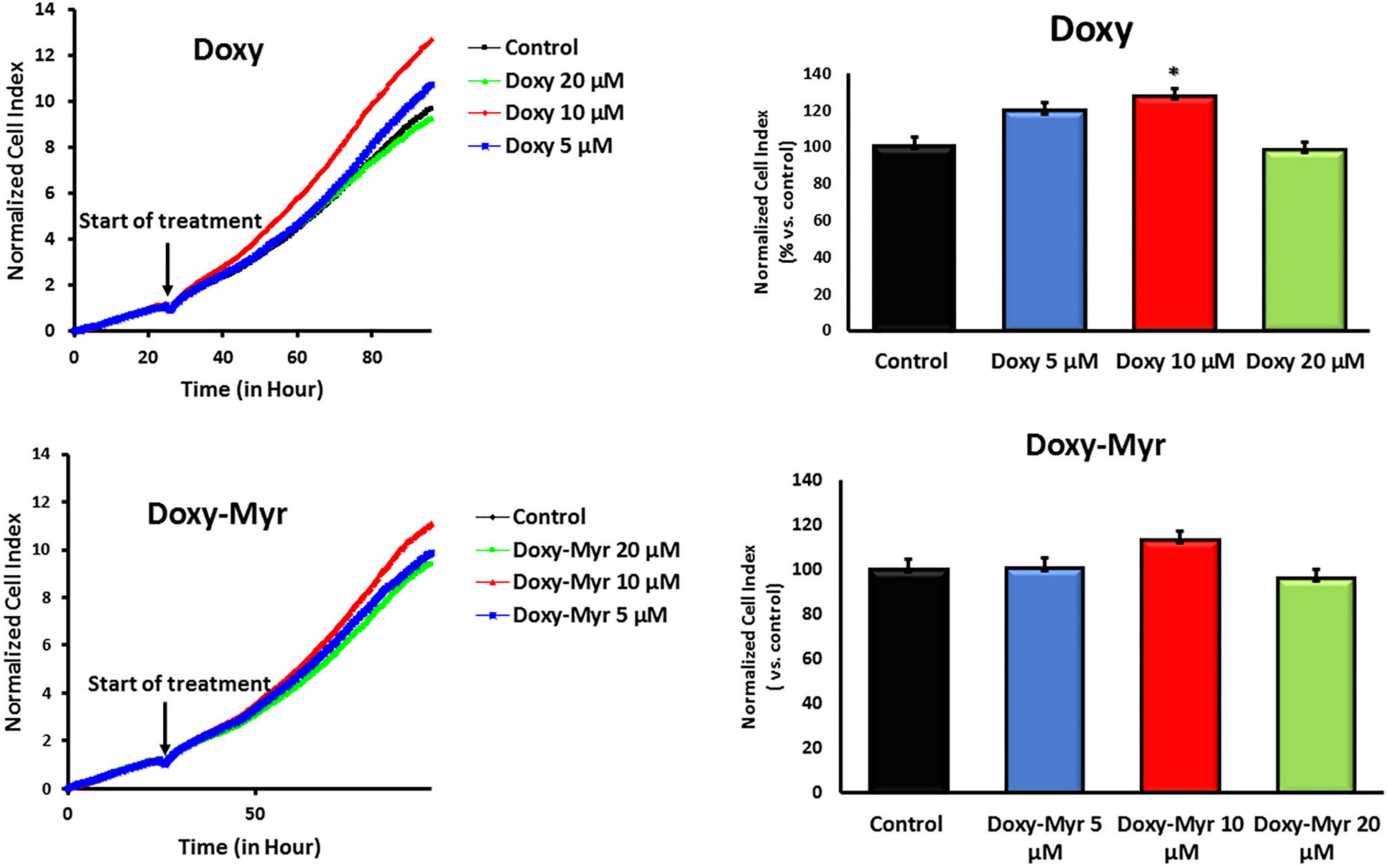
Doxy-Myr does not inhibit the proliferation of MCF7 cell 2D-monolayers. Potential effects of Doxycycline and Doxy-Myr on cell proliferation were determined using MCF7 cell monolayers. Note that Doxycycline and Doxy-Myr did not inhibit the proliferation of MCF7 cells, as assessed using the xCELLigence, in the concentration range of 5–20 μM. *p < 0.05.

**Figure 6:**
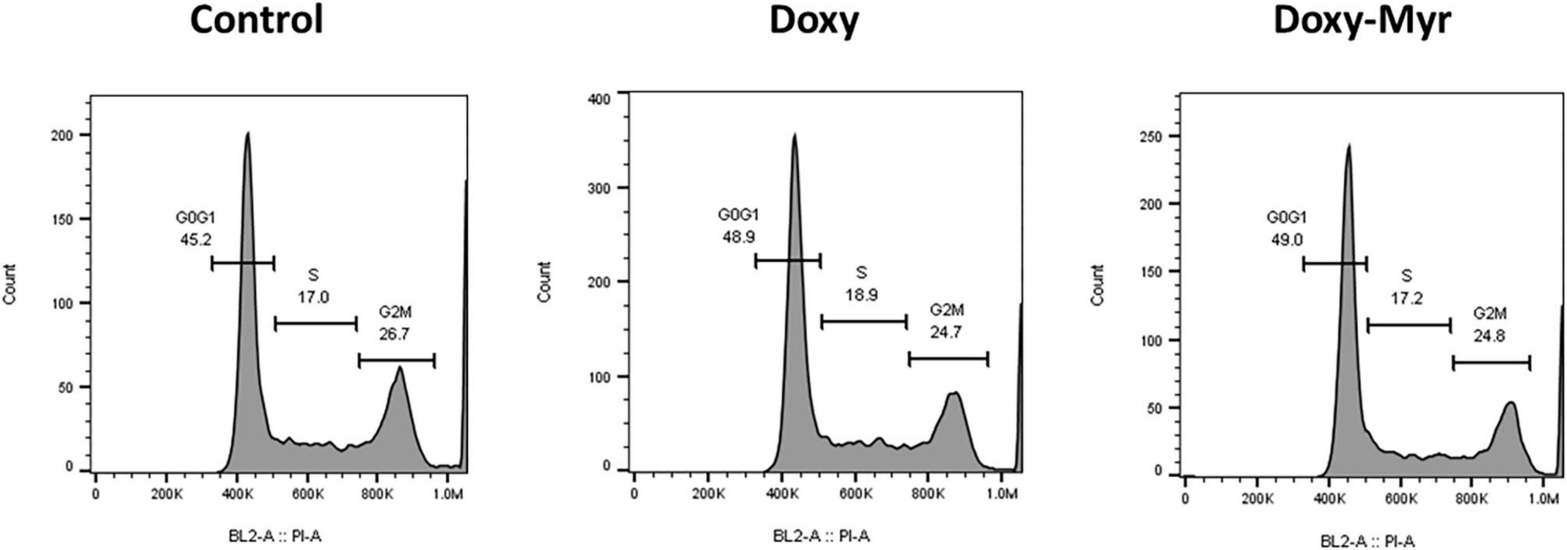
Doxy-Myr does not induce cell cycle arrest, in MCF7 2D-monolayers. MCF7 cells were cultured for 72 h as 2D-monolayers, in the presence of Doxycycline or Doxy-Myr, at a concentration of 10 μM. Vehicle-alone controls were processed in parallel. Note that relative to the parent compound Doxycycline, Doxy-Myr did not have any significant effects on reducing cell cycle progression in 2D-monolayers of MCF7 cells. Representative FACS cell cycle profiles are shown.

Potential 2D-effects on cell proliferation and the cell cycle were also determined using MCF7 cell monolayers. Clearly, Doxycycline and Doxy-Myr did not inhibit the proliferation of MCF7 cells, as assessed using the xCELLigence (Figure 5). Similarly, relative to the parent compound Doxycycline, Doxy-Myr did not have any significant effects on reducing cell cycle progression in 2D-monolayers of MCF7 cells (Figure 6).

Therefore, overall Doxy-Myr did not significantly reduce the viability, proliferation or cell cycle progression of 2D-monolayers of MCF7 cells, indicating that its effects were specific for cell propagation under 3D anchorage-independent growth conditions.

### Doxy-Laur and Doxy-Pal Are Less Potent Than Doxy-Myr in Targeting CSCs

**Figure 7:**
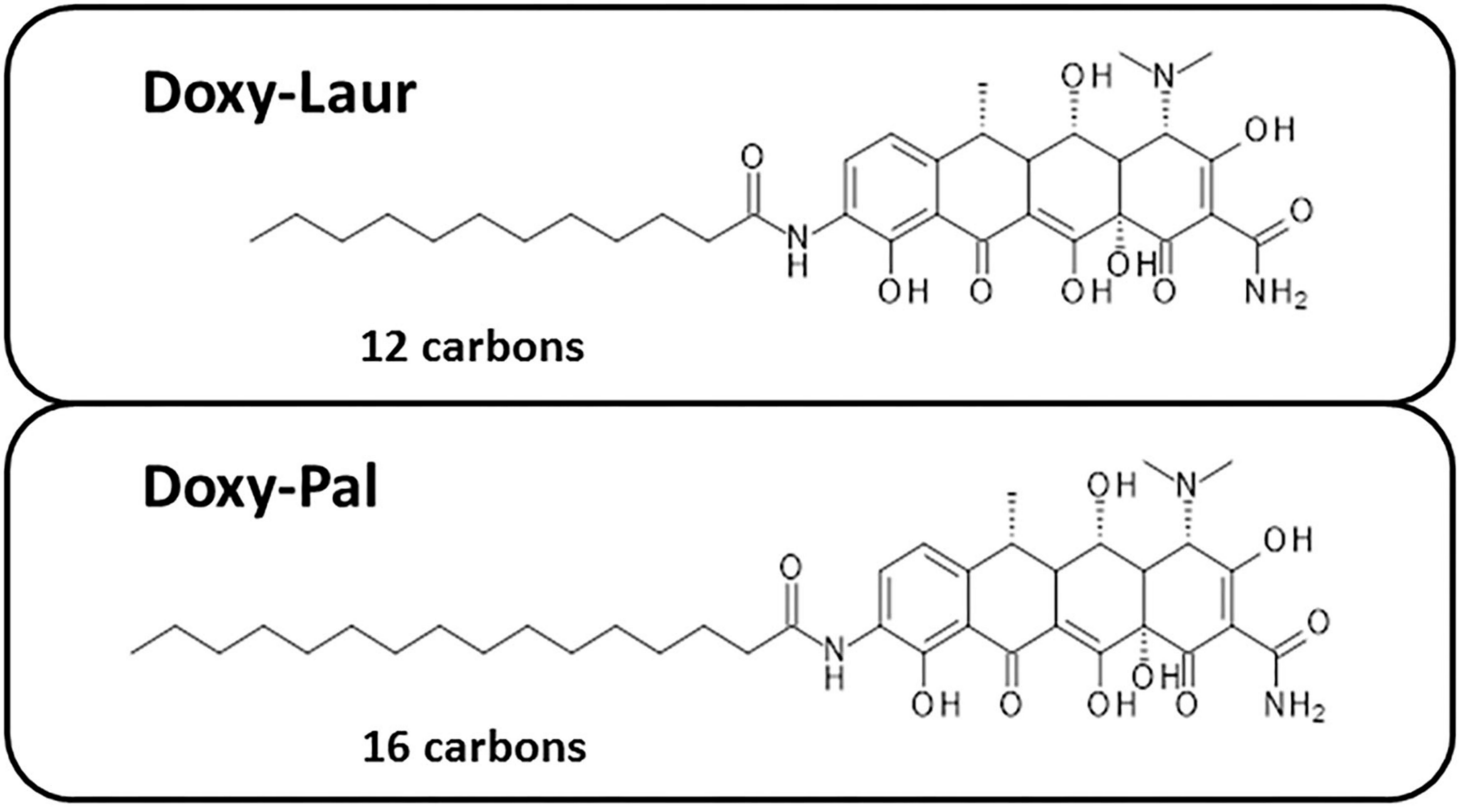
Chemical structures of two other Doxycycline derivatives: Doxy-Laur and Doxy-Pal. We created two other new Doxycycline analogs by varying the chain length of the fatty acid attached to 9-amino-Doxycycline. These two analogs included Doxy-Laur (harboring a 12 carbon fatty acid) and Doxy-Pal (harboring a 16 carbon fatty acid). Their chemical structures are as shown.

**Figure 8:**
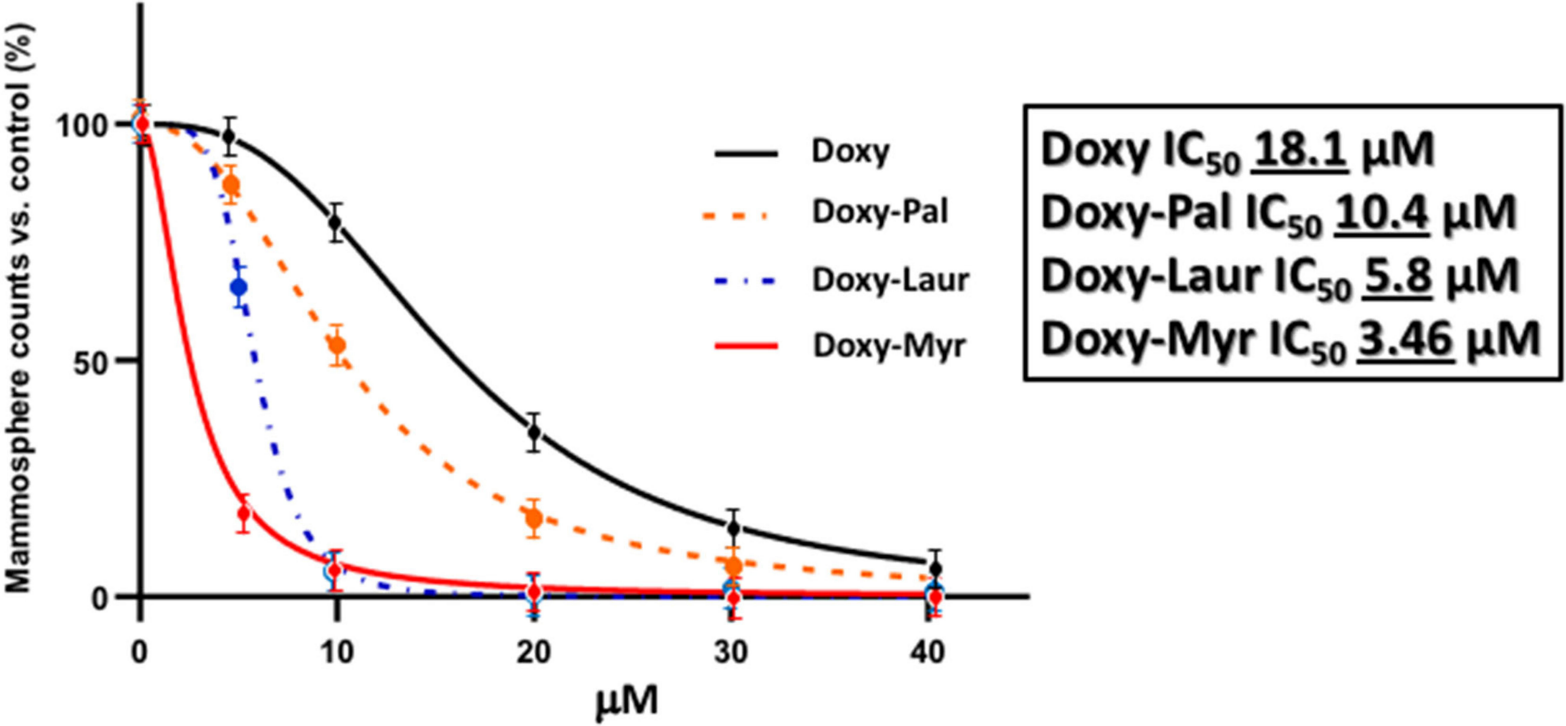
Rank order potency of the new Doxycycline derivatives. Note that Doxy-Myr is the most potent Doxycycline derivative for targeting CSC propagation, as assayed using the 3D mammosphere assay to quantitatively measure anchorage-independent growth. The rank order potency is Doxy-Myr > Doxy-Laur > Doxy-Pal > Doxycycline.

We also synthesized two other new Doxycycline analogs to study the influence of the fatty acid chain length on their functional activity. These two analogs included Doxy-Laur (harboring a 12 carbon fatty acid) and Doxy-Pal (harboring a 16 carbon fatty acid) (Figure 7). Therefore, we directly compared the functional inhibitory activity of Doxy-Myr, Doxy-Laur, and Doxy-Pal in the 3D-mammosphere assay, using MCF7 cells. Interestingly, Figure 8 demonstrates that the rank order potency is: Doxy-Myr > Doxy-Laur > Doxy-Pal > Doxycycline, with no direct correlation observed between chain length and activity. As such, the addition of myristic acid (a 14 carbon fatty acid) appears to be the optimal chain length modification.

### Doxy-Myr, Doxy-Laur, and Doxy-Pal Lack Antibiotic Activity Against Common Gram-Negative and Gram-Positive Bacteria

Doxycycline is a well-established broad-spectrum antibiotic, that is routinely used for therapeutically targeting both gram-negative and gram-positive bacterial infections. As a consequence, we also assessed the antibiotic activity of the Doxycycline analogs, as compared to the parent compound Doxycycline.

**Figure 9:**
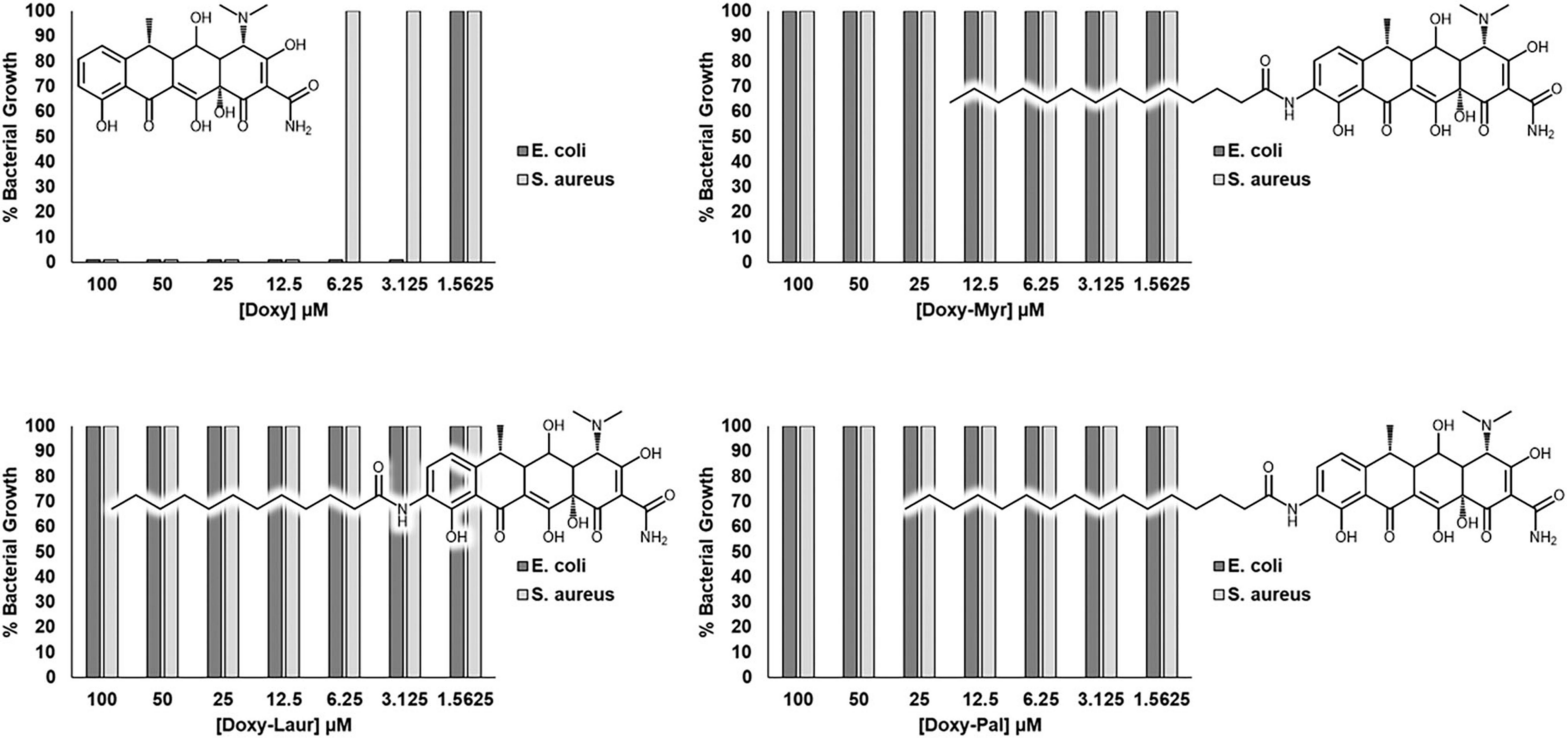
Doxy-Myr, Doxy-Laur and Doxy-Pal do not show any residual antibiotic activity. The novel Doxycycline analogs (Doxy-Myr, Doxy-Laur, and Doxy-Pal) were screened against a gram-positive (*S. aureus*; ATCC 29213) and a gram-negative (*E. coli*; ATCC 25922) strain of bacteria to verify the maintenance of the antibiotic activity, when compared to the parent compound. While Doxycycline presented MIC values of 3.125 μM against *E. coli* and 12.5 μM against *S. aureus*, none of the Doxycycline analogs inhibited bacterial growth up to the concentration of 100 μM.

Figure 9 reveals that, as expected, Doxycycline potently and effectively inhibits the growth of both gram-negative (*E. coli*) and gram-positive (*S. aureus*) micro-organisms. Minimum inhibitory concentrations were 3.125 μM (1.3 mg/L) and 12.5 μM (5.5 mg/L), respectively. However, in striking contrast, Doxy-Myr, Doxy-Laur, and Doxy-Pal did not show any obvious antibiotic activity, in the same concentration range. Minimum inhibitory concentrations of the Doxycycline analogs were >100 μM (>65 mg/L). The clinical breakpoints for tetracyclines against *E. coli* and *S. aureus* are between 0.5–2.0 mg/L and 2.0–6.0 mg/L, respectively (28).

Therefore, these simple chemical modifications of Doxycycline have successfully removed its ability to act as a functional antibiotic, while simultaneously increasing its specificity for targeting CSCs.

### Doxy-Myr Potently Inhibits Cancer Cell Metastasis *in vivo*, Without Significant Toxicity

To experimentally evaluate the functional effects of Doxycycline and Doxy-Myr *in vivo*, we next used MDA-MB-231 cells and the well-established chorio-allantoic membrane (CAM) assay in chicken eggs, to quantitatively measure tumor growth and metastasis (16–19). MDA-MB-231 breast cancer cells were used for our *in vivo* studies, as they are estrogen-independent, intrinsically more aggressive, form larger tumors and are significantly more migratory, invasive and metastatic. As such, they are a better *in vivo* model, for simultaneously evaluating both tumor growth and spontaneous metastasis. Moreover, we have previously demonstrated that Doxycycline also effectively inhibits the 3D anchorage-independent growth of MDA-MB-231 cells (13).

Briefly, an inoculum of 1 × 10^6^ MDA-MB-231 cells was added onto the Upper CAM of each egg (day E9) and then eggs were randomized into groups. On day E10, tumors were detectable and they were then treated daily for 8 days with vehicle alone (1% DMSO in PBS), Doxycycline or Doxy-Myr. After 8 days of drug administration, on day E18 all tumors were weighed, and the Lower CAM was collected to evaluate the number of metastatic cells, as analyzed by qPCR with specific primers for Human Alu sequences.

Morphologically, the CAM is constructed of two opposing sheets of epithelial cells, which are separated by a middle stromal layer, containing blood vessels and lymphatics. One epithelial layer is of ectodermal origin, while the other epithelial layer is of mesodermal/endodermal origin. Importantly, movement of metastatic MDA-MB-231 cells from the Upper CAM to the Lower CAM involves their migration away from the primary tumor, cellular invasion, intravasation, extravasation, and the formation of a new distant lesion, all of the normal steps that are key features of spontaneous tumor cell metastasis (see Supplemental Figure 2).

**Figure 10:**
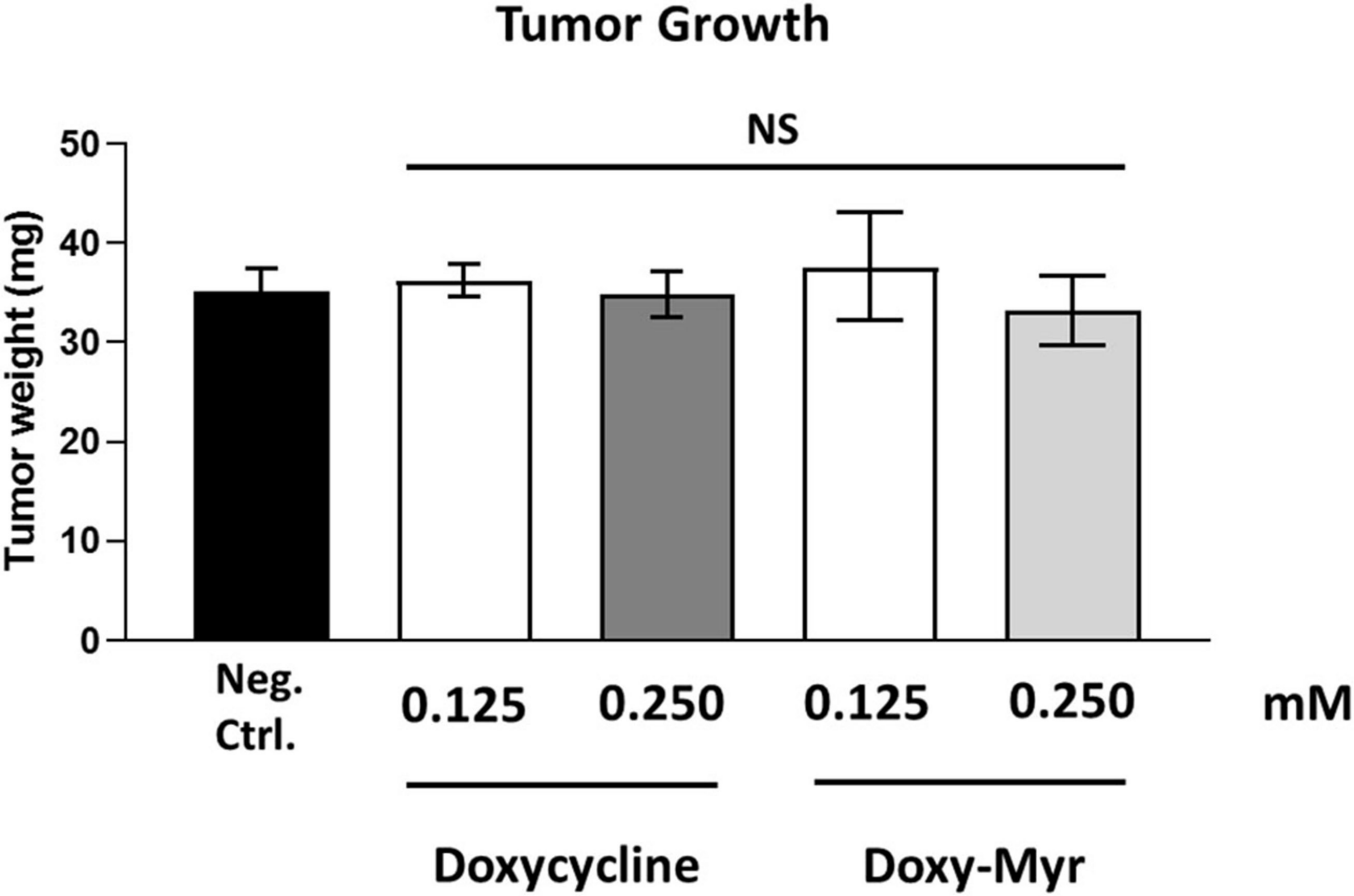
Doxycycline and Doxy-Myr have no effect on tumor growth. MDA-MB-231 cells and the well-established chorio-allantoic membrane (CAM) assay in chicken eggs were used to quantitatively measure tumor growth. An inoculum of 1 × 10^6^ MDA-MB-231 cells was added onto the Upper CAM of each egg (on Day E9) and then eggs were then randomized into groups. On day E10, tumors were detectable and they were then treated daily for 8 days with vehicle alone (1% DMSO in PBS), Doxycycline or Doxy-Myr. After 8 days of drug administration, on day E18 all tumors were weighed. Note that Doxycycline and Doxy-Myr did not have any significant effects on tumor growth. Averages are shown ± SEM. NS, not significant.

Figure 10 shows the effects of Doxycycline and Doxy-Myr on MDA-MB-231 tumor growth. Note that that they both did not show any significant effects on tumor growth, as a result of the 8-day period of drug administration.

**Figure 11:**
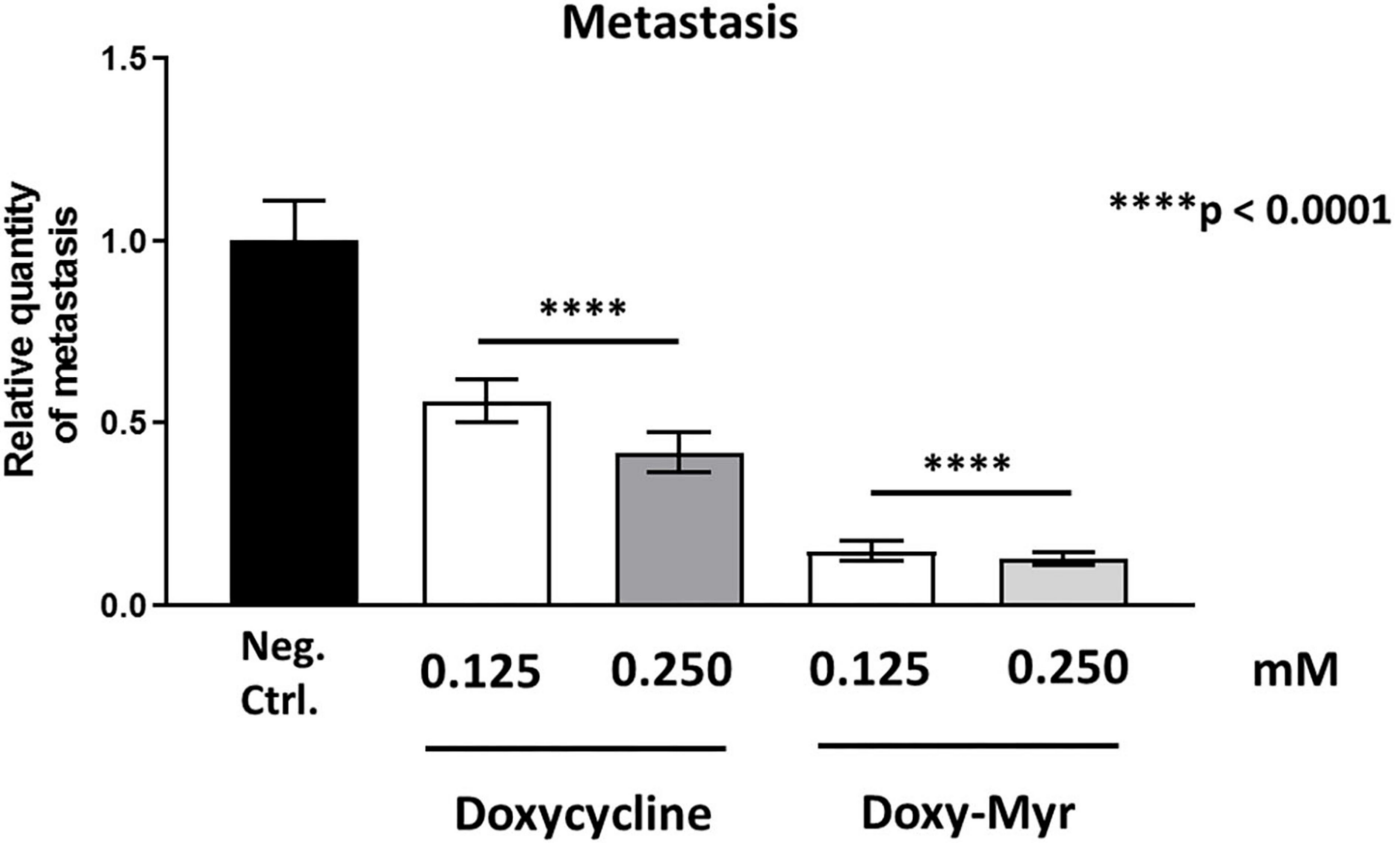
Doxycycline and Doxy-Myr selectively target and prevent cancer metastasis. MDA-MB-231 cells and the well-established chorio-allantoic membrane (CAM) assay in chicken eggs were used to quantitatively measure spontaneous tumor metastasis. An inoculum of 1 × 10^6^ MDA-MB-231 cells was added onto the Upper CAM of each egg (on day E9) and then eggs were then randomized into groups. On day E10, tumors were detectable and they were then treated daily for 8 days with vehicle alone (1% DMSO in PBS), Doxycycline or Doxy-Myr. After 8 days of drug administration, the Lower CAM was collected to evaluate the number of metastatic cells, as analyzed by qPCR with specific primers for Human Alu sequences. Note that Doxycycline and Doxy-Myr both showed significant effects on MDA-MB-231 metastasis. However, Doxy-Myr was clearly more effective than Doxycycline in inhibiting metastasis. Averages are shown ± SEM. ****p < 0.0001.

However, both Doxycycline and Doxy-Myr showed significant effects on MDA-MB-231 cancer cell metastasis. Figure 11 illustrates that Doxycycline inhibited metastasis (by 44–57.5%). In contrast, Doxy-Myr inhibited metastasis (by 85–87%), at the same concentrations tested. Interestingly, the effects of Doxy-Myr on metastasis were significantly more pronounced.

Surprisingly, little or no embryo toxicity was observed for Doxycycline and Doxy-Myr (Table 1). Therefore, we conclude that Doxy-Myr can be further developed as an anti-metastatic agent, selectively inhibiting tumor metastasis, without showing significant toxicity or antibiotic activity.

**Table 1:** Chick embryo toxicity of Doxycycline and Doxy-Myr.

**Group. #**	**Group description**	**Total**	**Alive**	**Dead**	**% Alive**	**% Dead**
1	Neg. Ctrl.	18	15	3	83.33	16.67
2	Doxy, 0.125 mM	13	11	2	84.62	15.38
3	Doxy, 0.250 mM	13	10	3	76.92	23.08
4	Doxy-Myr, 0.125 mM	14	11	3	78.57	21.43
5	Doxy-Myr, 0.250 mM	13	12	1	92.31	7.69

## Discussion

**Figure 12:**
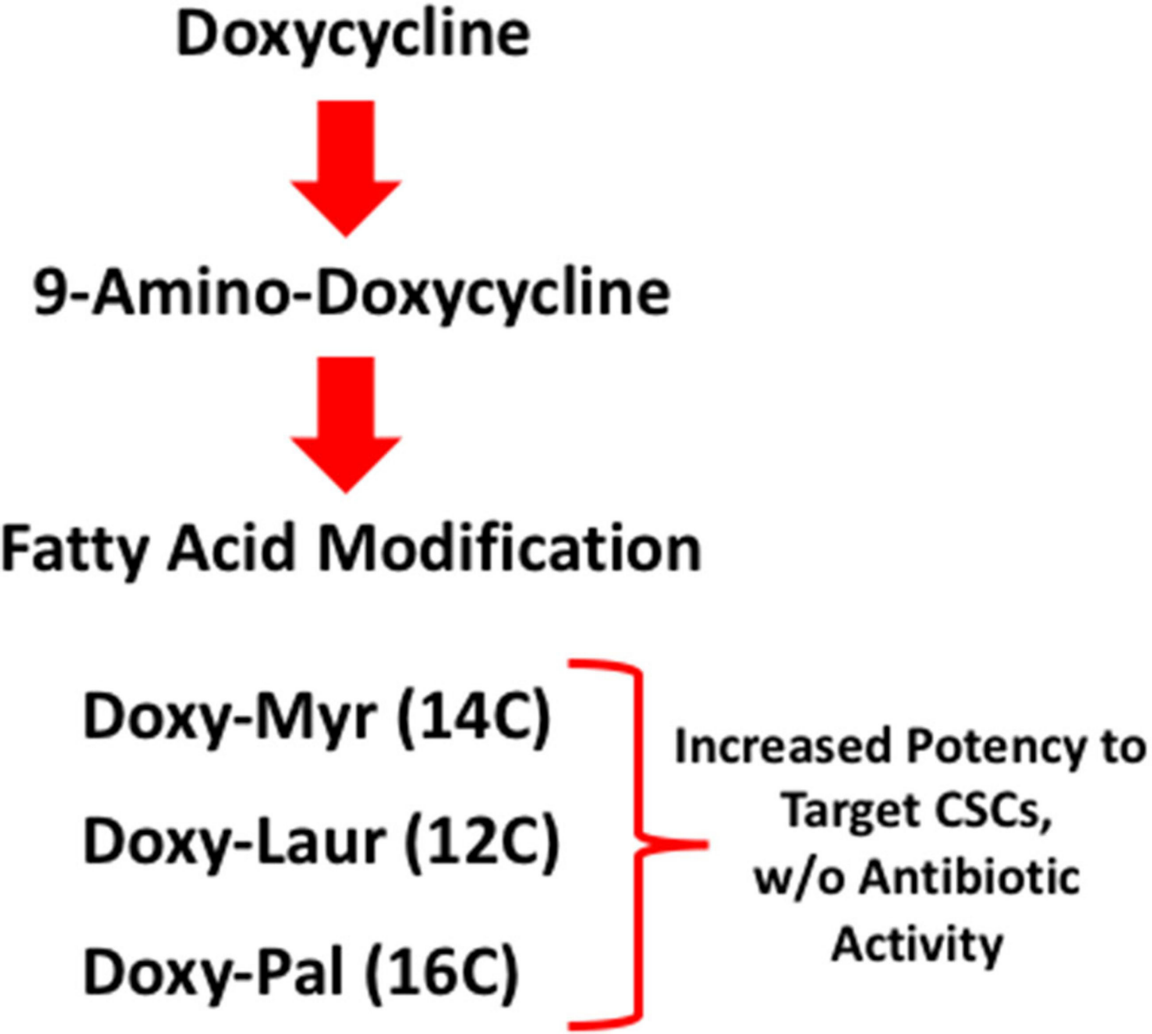
Schematic diagram highlighting our systematic approach to generating Doxycycline derivatives, to target CSCs and prevent metastasis. Lipid moieties were covalently conjugated to 9-amino-Doxycycline. Importantly, these three Doxycycline derivatives lack anti-microbial activity.

Here, we report the chemical synthesis and biological activity of several new Doxycycline analogs, modified to increase their effectiveness in the targeting of CSCs. The most promising compound was Doxy-Myr, a Doxycycline analog in which a myristic acid (14 carbon) moiety is covalently attached to the free amino group of 9-amino-Doxycycline. We analyzed the potency of Doxy-Myr, using the 3D-mammosphere assay, to assess its potential inhibitory effects on the anchorage-independent propagation of breast CSCs. Overall, we observed that Doxy-Myr is >5-fold more potent than Doxycycline, the parent compound. Moreover, Doxy-Myr showed better intracellular retention, and was specifically localized within a peri-nuclear membranous compartment. In striking contrast, when MCF7 breast cancer cells or normal fibroblasts were grown as 2D-monolayers, Doxy-Myr did not reveal any effects on cell viability or proliferation, highlighting its unique selectivity for targeting the 3D-propagation of CSCs. In addition, we evaluated other Doxycycline analogs, with longer (16 carbon; palmitic acid) or shorter (12 carbon; lauric acid) chain lengths (Figure 12). However, these two analogs were less effective than Doxy-Myr for targeting of CSCs. Finally, using MDA-MB-231 cells, we demonstrated that Doxy-Myr has no appreciable effects on tumor growth, but potently inhibits tumor cell metastasis *in vivo*, with little or no chick embryo toxicity.

Our results also showed that the lipophilic amide substituents in Doxycycline on the C9 of the Tetracycline (TC) skeleton led to the loss of its antibacterial activity. Previously published structure-activity relationship (SAR) studies have shown that chemical modification of the TC skeleton on C9 can be tolerated, leading to diverse antibacterial activity, as is exemplified by the antibiotic Tigecycline. The lipophilicity of the TCs seems to play a key role in the biological potency of this family of drugs. There is a trend of decreased antibiotic activity with the increase of lipophilicity, specifically against gram-negative species, with eventual loss of activity when high lipophilicity is achieved, which could partially explain the loss of activity, we observed after the addition of fatty acids to the Doxycycline scaffold.

Importantly, our improvement in the biological properties of these Doxycycline analogs for targeting CSCs and the associated loss of the anti-microbial activity, make these new analogs extremely promising, because tetracycline resistance among gram-negative and gram-positive pathogens requires exposure to inhibitory concentrations as a selective pressure (28–30). MICs exceeding 65 mg/L also suggest that these analogs might be non-inhibitory to members of the human microbiome, the complex community of microorganisms which exerts wide-ranging effects on human immunity and disease (29, 30).

Interestingly, a previous study successfully used the parent compound, Doxycycline, to prevent bone metastasis in a mouse model, by employing MDA-MD-231 cells (31). However, these authors did not examine the effects of Doxycycline on tumor growth, but only focused on bone metastasis. They attributed the efficacy of Doxycycline to its tropism for bone and to its ability to act as a protease inhibitor for lysosomal cysteine proteinases, the cathepsins, and MMPs, because Doxycycline behaves as a zinc chelator.

**Figure 13:**
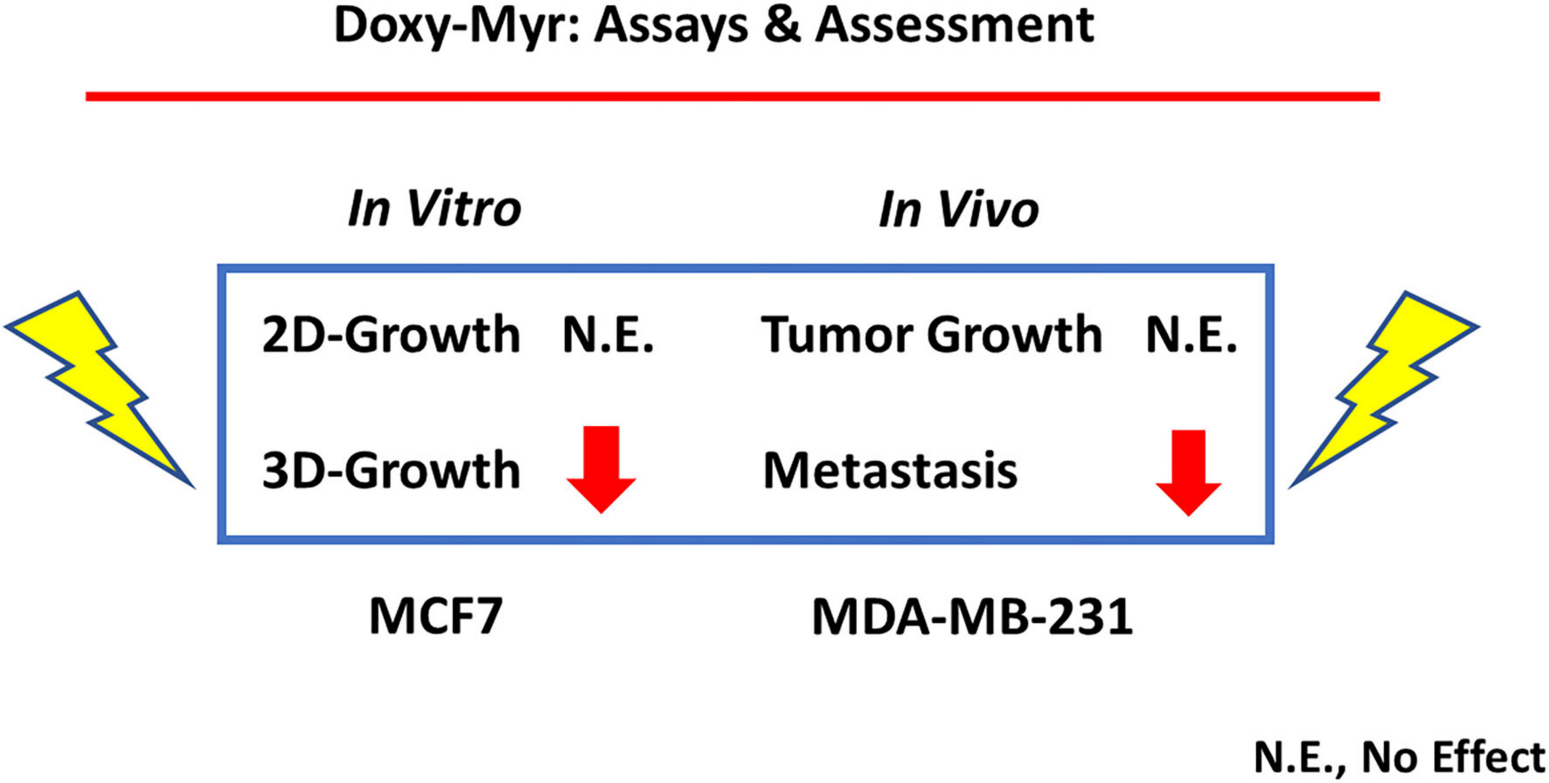
*In vitro* inhibition of 3D-growth predicts *in vivo* inhibition of metastasis. Here, we used two complementary breast cancer cell lines for our *in vitro* screening (MCF7) and *in vivo* (MDA-MB-231) validation assays. More specifically, Doxy-Myr had no effect on 2D-growth *in vitro* and no effect on tumor growth *in vivo*. Conversely, we showed that Doxy-Myr potently inhibited 3D-growth *in vitro*, which directly correlated with inhibition of metastasis *in vivo*. In support of this observation, 3D anchorage-independent growth is thought to be a required step for metastasis *in vivo*. N.E., no effect.

**Figure 14:**
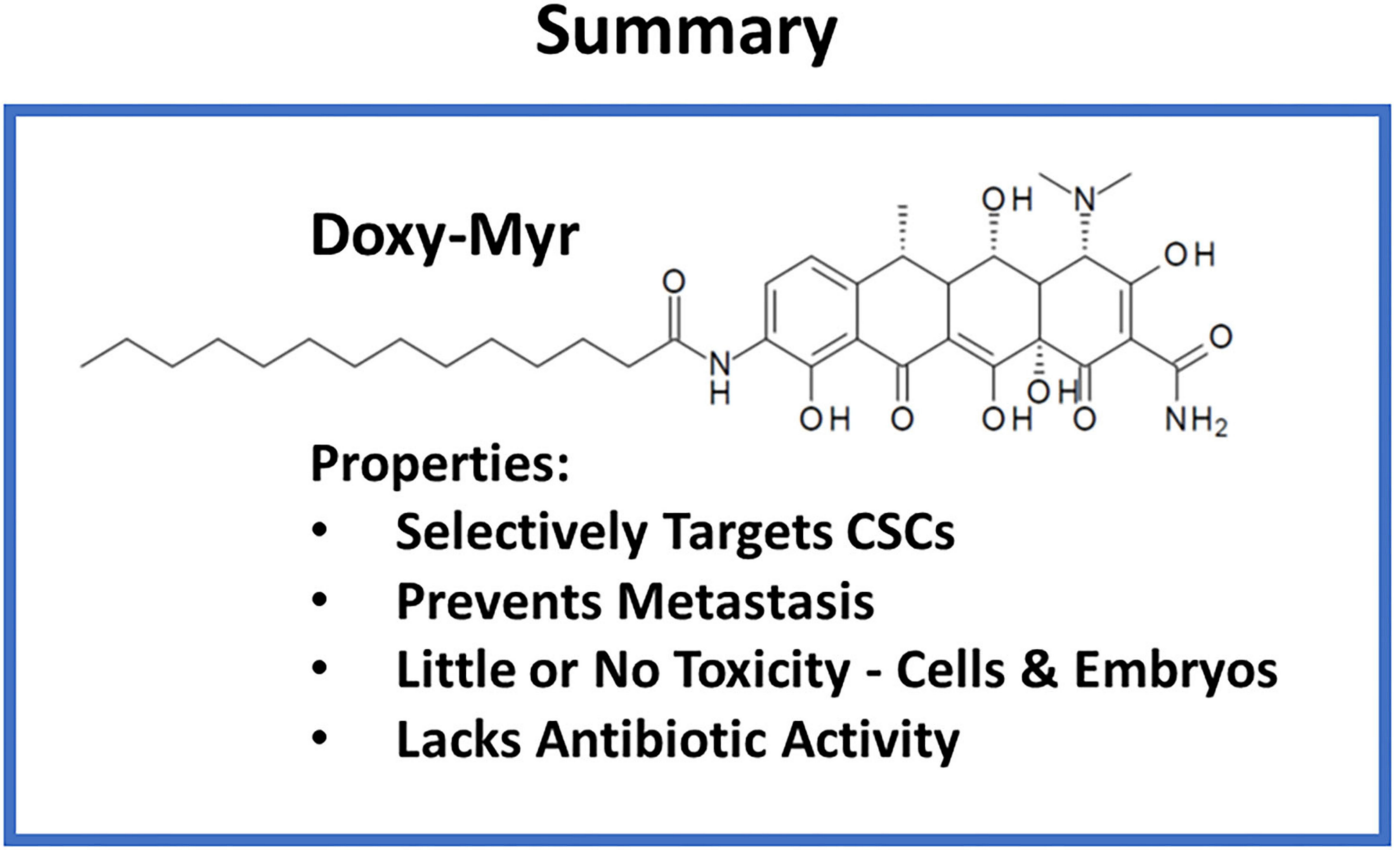
Summary: Properties of Doxy-Myr. Briefly, Doxy-Myr is a lipid modified Doxycycline derivative. Our results show that Doxy-Myr potently targets CSCs and selectively prevents metastasis, without affecting tumor growth. Moreover, Doxy-Myr was non-toxic in the chick embryo assay and did not affect the viability of normal cells, or MCF7 cells, grown as a 2D-monolayer. Importantly, Doxy-Myr lacked antibiotic activity, and did not affect the growth of gram-positive (*E. coli*) or gram-negative (*S. aureus*) organisms.

In contrast, herein, we have demonstrated that Doxycycline and Doxy-Myr both act as inhibitors of metastasis, by targeting the 3D anchorage-independent growth of CSCs, which is a completely different molecular mechanism (Figures 13, 14). However, as predicted, Doxy-Myr was significantly more effective than Doxycycline, at the same concentrations examined. As such, based on these functional observations, we propose that this overall lipid modification strategy may be generally applicable, to facilitate the development and discovery of other drugs, for effectively preventing tumor progression, recurrence, and distant metastasis.

Moreover, our current results are consistent with recent studies showing that prophylaxis with other classes of mitochondrial inhibitors is indeed sufficient to prevent metastasis, using the same pre-clinical xenograft model, with little or no effect on tumor growth and minimal toxicity (32).

## Data Availability Statement

All datasets generated for this study are included in the article/Supplementary Material.

## Author Contributions

ML and FS conceived and initiated this project, they selected the clinically-approved drug Doxycycline for chemical modification and optimization by medicinal chemistry. JK performed the custom-chemical syntheses. The phenotypic drug screening and the majority of other wet-lab experiments described in this paper were performed by BÓ. LM determined the antibiotic activity of Doxycycline and its derivatives. BÓ, JK, and LM generated the final figures for the paper. ML and FS wrote the first draft of the manuscript, which was then further edited and approved by BÓ, LM, JL, JK, FS, and ML. ML generated the schematic summary diagrams. All authors contributed to the article and approved the submitted version.

## Conflict of Interest

JK is employed by the company Eurofins Integrated Discovery UK Ltd. FS holds a part-time affiliation with Lunella Biotech, Inc. ML holds a part-time affiliation with Lunella Biotech, Inc. The remaining authors declare that the research was conducted in the absence of any commercial or financial relationships that could be construed as a potential conflict of interest.

## Supplementary Material

Supplemental Figure 1 Chemical synthesis of Doxycycline derivatives.

Supplemental Figure 2 CAM model for measuring tumor growth, metastasis, and embryo toxicity. On day E9, an inoculum of 1 million MDA-MB-231 breast tumor cells was layered on top of the Upper CAM and was allowed to form a primary tumor. Potential therapeutics were applied for a period of 8-days. Then, on day E18, the primary tumor was harvested from the upper CAM and the magnitude of distant metastases was quantitated in the Lower CAM, by performing qPCR with specific primers for recognizing Human Alu sequences. In order for the cells to metastasize, from the Upper CAM to the Lower CAM, it has been established that they need to undergo migration, invasion, intravasation, extravasation, and secondary lesion formation. Toxicity was measured by scoring embryo viability on day E18. See *Materials and Methods* for further details. Reproduced and modified, under a creative commons license, from the following source (33).
