# Supplementary material for: A Myristoyl Amide Derivative of Doxycycline Potently Targets Cancer Stem Cells (CSCs) and Prevents Spontaneous Metastasis, Without Retaining Antibiotic Activity
Source: Front Oncol. 2020 Sep 15;10:1528. doi: 10.3389/fonc.2020.01528 (PMC7523513; doi:10.3389/fonc.2020.01528)
Supplement: Supplementary file 1 [file Data_Sheet_1.PDF]

Supplemental Materials and Methods for the Chemical Synthesis of Doxycycline Derivatives

Doxycycline hydrate was purchased from AlfaAesar.

Doxycycline derivatives **1**-**4** were synthesized following the general method for (4S,5S,6R,12aS)-4-(dimethylamino)-3,5,10,12,12a-pentahydroxy-6-methyl-1,11-dioxo-9-(tetradecanoylamino)-4a,5,5a,6-tetrahydro-4H-tetracene-2-carboxamide (**1**).

Step (**a**): To a stirred solution of doxycycline hydrate (1.0g, 2.16mmol) in conc. H_2SO_4 (5.5ml) at room temperature under nitrogen atmosphere $NaNO_3$ (0.29g, 3.41mmol) was added and the mixture was stirred for 3 hours. The resulting dark brown oil was poured into ice cold diethyl ether (140ml), the precipitate was collected under nitrogen atmosphere, washed with diethyl ether and dried under vacuum to yield a crude 9-nitrodoxycycline.

Step (**b**): The crude 9-nitrodoxycycline (1.0g, 2.04mmol) was dissolved in methanol (30ml) at room temperature under nitrogen atmosphere, PtO_2 (0.12g) was added and the suspension was stirred under hydrogen atmosphere for 2 hours. The catalyst was removed by filtration through Celite pad, the filtrate was poured into diethyl ether (240ml) under nitrogen atmosphere and the precipitate was collected and dried under vacuum to yield a crude 9-aminodoxycycline (0.89g, 1.94 mmol).

Step (**c**): The crude 9-aminodoxycycline (0.70g, 1.5mmol), myristic acid (0.36g, 1.5mmol), HBTU (0.85g, 2.25mmol) and NMM (0.33ml, 3.0mmol) in a mixture of DCM (12ml) and DMF (4ml) was stirred under nitrogen atmosphere at room temperature for 72 hours. The solvents were evaporated under reduced pressure. The resulting residue was triturated with acetonitrile (40ml), the precipitation was collected by filtration, was washed with acetonitrile (10ml), diethyl ether (20ml) and dried under vacuum. The crude product was dissolved in DMSO and purified by preparative HPLC to yield (4S,5S,6R,12aS)-4-(dimethylamino)-3,5,10,12,12a-pentahydroxy-6-methyl-1,11-dioxo-9-(tetradecanoylamino)-4a,5,5a,6-tetrahydro-4H-tetracene-2-carboxamide (**1**), (0.086g). ^{1}H-NMR (MeOD)δ 0.89 (dd, 3H), 1.14-1.48 (m, 20H), 1.54 (d, 3H), 1.61-1.79 (m, 2H), 2.38-2.53 (dd, 2H), 2.49-2.61 (m, 2H), 2.65 (m, 8H), 3.68 (dd, 1H), 3.94 (m, 1H), 6.93 (d, 1H), 8.14 (d, 1H). LC-MS 670.2 $[M+H]^+$, RT 2.78min.

(4S,5S,6R,12aS)-4-(dimethylamino)-9-(dodecanoylamino)-3,5,10,12,12a-pentahydroxy-6-methyl-1,11-dioxo-4a,5,5a,6-tetrahydro-4H-tetracene-2-carboxamide (**2**). LC-MS 642.1 $[M+H]^+$, RT 2.42min.

(4S,5S,6R,12aS)-4-(dimethylamino)-9-(hexadecanoylamino)-3,5,10,12,12a-pentahydroxy-6-methyl-1,11-dioxo-4a,5,5a,6-tetrahydro-4H-tetracene-2-carboxamide (**3**).LC-MS 698.2 $[M+H]^+$, RT 3.02min.

[6-[[(5R,6S,7S,10aS)-9-carbamoyl-7-(dimethylamino)-1,6,8,10a,11-pentahydroxy-5-methyl-10,12-dioxo-5a,6,6a,7-tetrahydro-5H-tetracen-2-yl]amino]-6-oxo-hexyl]-triphenyl-phosphonium oxalate (**4**). LC-MS 409.7 $[M ½]^+$, RT 1.53min.
