# Supplementary figures and images for: A Myristoyl Amide Derivative of Doxycycline Potently Targets Cancer Stem Cells (CSCs) and Prevents Spontaneous Metastasis, Without Retaining Antibiotic Activity
Source: Front Oncol. 2020 Sep 15;10:1528. doi: 10.3389/fonc.2020.01528 (PMC7523513; doi:10.3389/fonc.2020.01528)

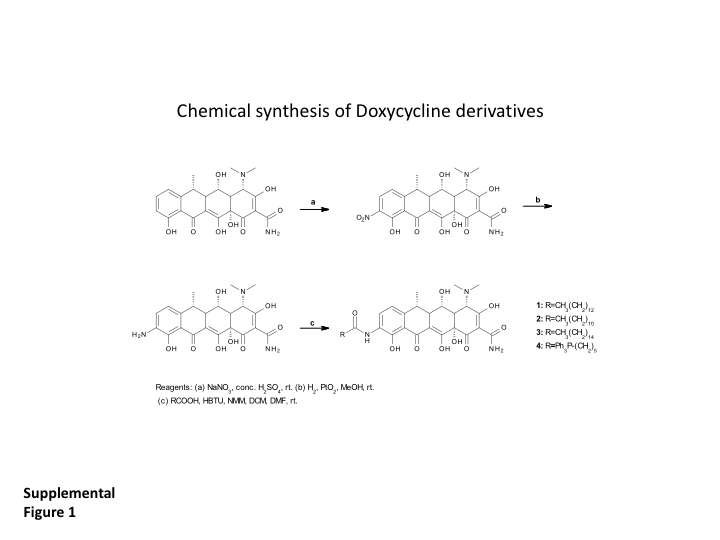

Supplement: Supplemental Figure 1 — Chemical synthesis of Doxycycline derivatives. [file Image_1.tiff]

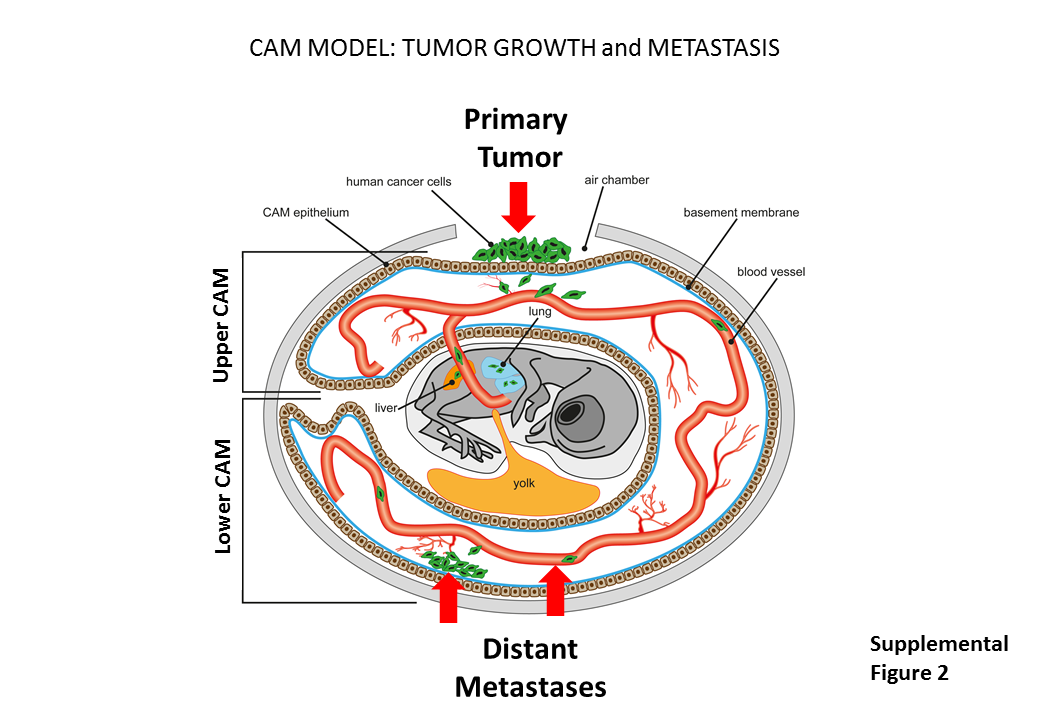

Supplement: Supplemental Figure 2 — CAM model for measuring tumor growth, metastasis, and embryo toxicity. On day E9, an inoculum of 1 million MDA-MB-231 breast tumor cells was layered on top of the Upper CAM and was allowed to form a primary tumor. Potential therapeutics were applied for a period of 8-days. Then, on day E18, the primary tumor was harvested from the upper CAM and the magnitude of distant metastases was quantitated in the Lower CAM, by performing qPCR with specific primers for recognizing Human Alu sequences. In order for the cells to metastasize, from the Upper CAM to the Lower CAM, it has been established that they need to undergo migration, invasion, intravasation, extravasation, and secondary lesion formation. Toxicity was measured by scoring embryo viability on day E18. See Materials and Methods for further details. Reproduced and modified, under a creative commons license, from the following source (33). [file Image_2.TIF]
